# The EBMT Immune Effector Cell Nursing Guidelines on CAR-T Therapy: A Framework for Patient Care and Managing Common Toxicities

**DOI:** 10.1007/s44228-022-00004-8

**Published:** 2022-07-08

**Authors:** Rose Ellard, Michelle Kenyon, Daphna Hutt, Erik Aerts, Maaike de Ruijter, Christian Chabannon, Mohamad Mohty, Silvia Montoto, Elisabeth Wallhult, John Murray

**Affiliations:** 1 The Royal Marsden Hospitals NHS Foundation Trust, Fulham Road, London, SW3 6JJ UK; 2 Kings College Hospital NHS Foundation Trust, London, UK; 3 The Edmond and Lily Safra Children’s Hospital, Jerusalem, Israel; 4 University Hospital Zurich, Zurich, Switzerland; 5 Amsterdam UMC Location VUmc, Amsterdam, The Netherlands; 6 Institut Paoli-Calmettes, Marseille, France; 7 Sorbonne University, INSERM, Saint-Antoine Hospital, Paris, France; 8 Barts Health NHS Trust, London, UK; 9 Sahlgrenska University Hospital, Göteborg, Sweden; 10 Christie Hospital NHS Foundation Trust, London, UK

**Keywords:** CAR-T, CAR-T therapy, Cellular therapy, Immunotherapy, Nursing, Nursing education

## Abstract

Chimeric antigen receptor T-cell (CAR T) therapy is a new and rapidly developing field. Centers across the world are gaining more experience using these innovative anti-cancer treatments, transitioning from the ‘bench’ to the ‘bedside’, giving benefit to an increasing number of patients. For those with some refractory hematological malignancies, CAR-T may offer a treatment option that was not available a few years ago.

CAR-T therapy is an immune effector cell and precision/personalized medicine treatment which is tailored to the individual patient and associated with a variety of unique adverse events and toxicities that necessitate specialist nursing/medical vigilance in an appropriate clinical setting. Subtle unrecognized signs and symptoms can result in rapid deterioration and, possibly, life threatening cardiorespiratory and/or neurological sequelae.

These guidelines have been prepared for nurses working in cellular therapy in inpatient, outpatient and ambulatory settings. Many nurses will encounter cellular therapy recipients indirectly, during the referral process, following discharge, and when patients are repatriated back to local centers. The aim of these guidelines is to provide all nurses with a practice framework to enable recognition, monitoring and grading of CAR-T therapy-associated toxicities, and to support and nurse these highly complex patients with confidence.

They have been developed under the auspices of several bodies of the European society for Blood and Marrow Transplantation (EBMT), by experienced health professionals, and will be a valuable resource to all practitioners working in cellular therapy.

## Introduction

Immunotherapy using genetically engineered T cells that express a chimeric antigen receptor (CAR-T) is a relatively new technology in cancer treatment, the use of which has expanded rapidly in the field of malignant hematological diseases. CAR-T cells are autologous (self) when using the patient’s own T cells or allogeneic (from a donor), either derived from a human leukocyte antigen (HLA)-matched donor following a stem cell transplant (SCT), or from a non-matched healthy donor in the form of an ‘off the shelf’ and possibly ‘universal’ product, where the endogenous T-cell receptor is edited out [1]

This paper aims to provide all nurses with a practice framework to enable recognition, monitoring and grading of CAR-T therapy-associated toxicities, and to support and nurse these highly complex patients with confidence. The optimal care of patients undergoing CAR-T requires a multidisciplinary team approach, which includes nursing expertise. Nurses are key to the patient pathway and are involved in patient education, coordination, monitoring, escalation and treatment.

### How Does It Work?

T-cells are separated from blood mononuclear cells collected from patients or healthy donors, and are genetically modified to express the artificial receptor. The latter combines the extracellular domain of an immunoglobulin heavy chain (a fraction of an antibody), with the intracellular domain of the T-cell receptor that triggers T-cell activation and cytotoxicity upon tumor antigen binding. Patients receive conditioning treatments prior to CAR-T cell infusion, removing their immunosuppressive cells, thereby enabling in vivo CAR-T cell expansion. Ideally, the target antigen is expressed selectively on tumor cells with minimal expression on normal tissue. This, in turn, limits the damage to normal tissue, enhancing the overall effectiveness of this approach.

Treatment indication for CAR-T cell therapy is dependent on the criteria for the commercial product or clinical trial, but the following principles apply:

- Disease and remission criteria are according to published guidelines for specific indications, agreed criteria for individual CAR-T product or as per trial protocol. All cases should be discussed at a transplant Multi-Disciplinary Team Meeting or equivalent.
- Organ function assessment as per trial protocol or as per the requirements of the individual CAR-T product, usually similar to autologous stem cell transplant work up.
- Negative pregnancy test in women of childbearing age.
- Importance of barrier contraception should be discussed with all patients
- Baseline central nervous system (CNS) imaging and/or a lumbar puncture < 4 weeks prior to CAR-T infusion, to rule out CNS involvement may be required
- Patients usually need to have recovered from residual toxicities of previous treatments, including the resolution or absence of graft-versus-host disease

There are a number of commercially available products with further ones in development and others are being investigated in clinical trials. The CAR-T pathway is complex, and consists of a number of key steps including apheresis, manufacturing, possible cytoreduction/bridging therapy, conditioning chemotherapy, infusion, initial monitoring and follow up.

#### Nursing Care Prior to CAR-T Cell Administration

Indications for CAR-T cell treatment are evolving but, to date, most patients have been treated at a relatively advanced stage of disease, or with relapsed or refractory disease that has not responded to other standard or experimental treatments. This may contribute to anxiety, and many patients may be socially isolated, due to the significant distance from established support networks, if they are not treated at their local center. It is vital that the nurse and wider multi-disciplinary team are aware of these factors. Referral to counselling/psychology services at the treating center or local hospital should be offered, where appropriate. Given that these patients are likely to be refractory to a number of lines of therapy, and have an uncertain prognosis, it may also be appropriate to consider referral to palliative care services, to discuss advanced care planning and symptom control.

Nurses should ensure that the patient and family are provided with clear information as to the required evaluations to determine eligibility for CAR-T, including the assessment of disease status, blood tests, procedures (including pre- and post- procedure instructions); education as to the need for prophylactic medications, instructions for intravenous line care and potential complications from central lines. This list is not exhaustive.

Reducing the risk of complications prior to infusion is of paramount importance:

- Infection clearance and prophylaxis
  o Ensure the patient is free of active bacterial infection.
  o Virology testing to ensure no underlying active viral infections, such as Cytomegalovirus (CMV), Epstein-Barr virus (EBV), Human Herpes Virus 6 (HHV-6), HIV, Hepatitis B, C & E and adenovirus
  o Prophylactic antiviral, antifungal and pneumocystis pneumonia medications as per local policy
  o Covid 19 testing as per local policy
- Cytoreduction/bridging therapy
  o Consideration should be given to ensuring the patient has a low burden of disease, due to the risk of tumor lysis syndrome during chemotherapy, and an increased risk of cytokine release syndrome in the presence of a high disease burden. Some patients may require bridging therapy in the period between apheresis and infusion, as the manufacturing process can take a number of weeks. Tumor lysis prophylaxis may also need to be considered.
- Central venous access
  o Consideration of peripherally inserted central catheter (PICC) or central venous access line for intravenous fluids and other infusions.

Additional blood tests and investigations may be required as per local policy, trial protocol and/or the requirements of the CAR-T product.

## Apheresis

In order to produce autologous CAR-T cells, non-mobilized mature lymphocytes are collected from the peripheral blood via apheresis of mononuclear cells (MNC) [2]. Apheresis procedures are considered safe; however, the patient requires close monitoring of vital signs prior to and throughout the procedure. Good venous access is essential, and a vein assessment should be carried out prior to the procedure. Poor venous access and metabolic complications due to citrate toxicity are the main complications in apheresis [3]. Citrate toxicity symptoms during the apheresis procedure must be promptly recognized and treated immediately. Classically, symptoms are perioral numbness, paresthesia of the hands and feet, muscle cramps; nausea and vomiting. Calcium supplement by intravenous or oral routes may be required. Blood tests should be performed as per local apheresis unit guidelines.

The procedure with the most similarities to T-cell collection for CAR-T cell manufacture is MNC apheresis for donor lymphocyte collection. In both procedures, the target cells are mature T cells. However, lymphocyte donors are healthy with normal white blood cell and lymphocyte counts [4]. T cell collection for CAR T manufacturing is therefore technically more challenging, as patients are heavily pretreated and often have low leukocyte and lymphocyte counts [5]. Process specifications are manufacturer dependent, including shipment of fresh versus cryopreserved collected cell product.

The timing of apheresis is critical and should be closely coordinated, to take place after an appropriate washout period from previous systemic anti-cancer treatment, and prior to any further bridging chemotherapy. This is especially challenging for patients with relapsed progressive disease with a high blast count or disease burden. Steroids should not be used 7 days prior to apheresis. A further logistical challenge of the timing of apheresis is the availability of a manufacturing slot, manufacturing capacities are likely to rapidly improve.

Apheresis is a specialist area within hematology, and nurses working in this area require different knowledge and skills to those working in other areas.

## Pediatric Apheresis Considerations

The management of children can prove more challenging due to their physiology and small extracorporeal volume. The extracorporeal volume of the cell separator device is static. The smaller volume in low weight children (< 15–20 kg) may necessitate blood priming of the cell separator depending on institutional policy. Additionally, in children, the slow inlet rates that are required can lead to delays in establishing and maintaining a stable interface, increasing both total volumes processed and procedure time. Different centers have various protocols regarding venous access for apheresis. Some pediatric patients may require a leukapheresis catheter for cell collection [6]. In low body weight children, abdominal pain and restlessness may be the first and only signs of citrate toxicity.

## Nursing Care During CAR-T Cell Administration

### Infusion

CAR-T cells should be administered by nurses competent in immune effector cell therapy. The infusion should be scheduled during working hours to ensure a trained and experienced medical team is available in the event of an immediate adverse reaction.

Cell infusion is as per local policy and/or trial protocol, but the following principles should be observed:

- Check patient identity as per local policy (at all steps of the supply chain and patient care, maintain chain of custody / chain of identity)
- Explain the procedure to the patient
- Verify consent has been obtained
- Check prescription is correct
- Check vital signs and document.
  o Ensure patient is hemodynamically stable and apyrexial.
- Ensure all mandated pre-infusion assessments are complete, including a sample of the patient’s handwriting.
- Verify patent IV access.
- Ensure bedside emergency equipment (suction/oxygen) is in full working order. Prepare trolley with IV fluids and fresh IV line, to be used in the event of a reaction during infusion.
- Administer pre-medications as per prescription, this should not include steroids.
- Infuse cells as per local standard operating procedure/trial protocol, taking care to ensure that infusion takes place immediately post thawing with the correct giving set, as per the product requirements. Refer to manufacturer’s guidelines for further details.
- Observe for infusion-related reactions and escalate as per local policy.
- Ensure all necessary documentation is completed. CAR-T cells administered as part of a clinical trial will likely have additional documentation.

During scheduling of CAR-T cell infusion, inform the intensive care outreach team and neurology services of the planned infusion date.

## Corticosteroids

Cortico-steroids suppress T-cell function and/or induce T-cell apoptosis, and should be avoided for indications such as a pre-medication for blood products or for treatment of an allergic reaction. A steroid washout period is required prior to apheresis and CAR-T infusion; the manufacturer should advise on timelines for individual products.

## Supportive Care

Conditioning chemotherapy (lymphodepletion) can lead to prolonged (> 1–2 weeks) bone marrow suppression. Patients should receive anti-infective prophylaxis and other supportive care as per institutional autologous stem cell transplantation guidance. Suggested prophylactic medications may include.

- Levofloxacin
- Aciclovir
- Fluconazole
- Co-trimoxazole
- Lansoprazole
- Consider levetiracetam for patients at high risk of neurological toxicity
- Tumor lysis prophylaxis as per standard protocols

## Management of Infusion Reactions

Infusion reactions are managed as per local policy, trial protocol or manufacturer’s guidance; however, the following principles should be observed:

- Increase monitoring of vital signs
- Treat symptoms
- Only consider corticosteroids in a life-threatening scenario (though highly unlikely at this stage) and authorized by a senior clinician, due to their potential effect on the efficacy of CAR-T cells
- Ensure patient comfort and provide information and reassurance
- Document as per trial protocol/local policy

## Nursing Care After CAR-T Cell Administration

Nursing assessment:

- Inpatients should have strict 4 hourly vital signs recorded and documented throughout admission.
- ECG whilst an inpatient (frequency as per local policy).
- Daily blood tests which may include:
  o full blood count
  o renal function
  o liver function
  o coagulation
  o CRP
  o LDH
  o ferritin
  o cytokine levels (if available in treating center lab or as per trial protocol).
- Weekly virology blood panel as per treating center policy, and may include CMV, HHV6, adenovirus and EBV.
- Maintain accurate fluid balance chart with input and output records
- Weigh daily if inpatient, at every visit if outpatient.
- Daily handwriting tests as per Immune Effector Cell Associated Encephalopathy (ICE) tool (see below)

## Toxicities

CAR-T cell therapy bears significant acute and chronic toxicities ranging from mild to lethal. Three key toxicities require acquisition of expert nursing knowledge and care, as they are rarely experienced in other contexts:

- Cytokine Release Syndrome (CRS)
- Immune effector Cells Associated Neurotoxicity Syndrome (ICANS)
- Hemophagocytic lymphohistiocytosis (HLH)/macrophage activation syndrome (MAS)

## Cytokine Release Syndrome (CRS)

The most common acute toxicity following CAR-T administration is CRS. This can occur at any time between day 0 and day 14. Symptoms range from mild, with fever, malaise, hypotension and flu-like symptoms; to severe, with multi organ failure. The severity of CRS is dependent on tumor burden, intensity of lymphodepletion, proliferation rate and cytotoxicity of the CAR-T cell product [7].

CRS is driven by the activation of CAR-T cells, which release effector cytokines, such as IFNγ, TNFα, and IL-2. Increased IL-6 levels are associated with key clinical features of CRS, such as fever, hypoxia, hypotension and organ system failure [8].

The symptoms of CRS can mimic neutropenic sepsis. As such, the patient will need to be treated with broad spectrum antimicrobials. Further measures to treat CRS are described below. Patients and their care givers should be offered reassurance and education on the symptoms of CRS, and the reason for increased monitoring.

Patients who are being treated in the outpatient setting should receive comprehensive education on the symptoms of CRS and should attend the treating hospital without delay if they begin to feel unwell.

All patients should be monitored closely for signs of CRS for at least a month post CAR-T infusion, or as clinically indicated if complications arise.

Some key symptoms and blood test abnormalities to observe for, which may indicate development of CRS are:

- Fever and/or flu-like symptoms
- Hypotension
- Hypoxia
- Tachycardia or bradycardia
- Shortness of breath
- Vomiting more than once or diarrhea more than 4 times per day
- Rise in AST/ALT, bilirubin or CRP
- Decreasing urine output or acute kidney injury
- Blood clotting abnormalities with evidence of disseminated intravascular coagulation

Many of the above have other causes, (e.g. infection or drug toxicity); however, early identification of patients with CRS is essential, and all symptoms should be reported to the medical team. The life-threatening nature of this toxicity is one of the reasons why a tight and specific intra-hospital organization is mandated.

## Grading of CRS

Several different grading scales have been devised for CRS. The difference in grading scales used in different clinical trials has made comparison of toxicities between trials and products challenging.

In June 2018, the American Society for Transplantation and Cellular Therapy (ASTCT) met to harmonize definitions and grading systems for immune effector cell-associated CRS and neurotoxicity seen after immune effector cell therapies including CAR-T cells [9]. The ASTCT consensus grading of CRS, focuses on the symptoms of fever, hypotension and hypoxia. The published grading algorithm is reproduced in Table 1.

## Treatment of CRS

First line treatment for CRS is tocilizumab, a monoclonal antibody which binds to the IL-6 receptor and is licensed in Europe for treating CRS. The recommended dose is 8 mg/kg; with a maximum dose of 800 mg. It is given as an intravenous infusion over 60 min. Up to four doses can be given, at intervals of at least eight hours.

Second line treatment for CRS is usually steroids. The dose and choice will depend on the guidance provided by the product manufacturer and the caution should be exercised treating CRS with steroids, as their use can reduce the persistence and efficacy of CAR-T cells [7]. It is recommended that steroids are prescribed by a senior member of the medical team.

Other CRS treatments are in development and clinical data are currently limited.

## Neurological Toxicity: Immune Effector Cell Associated Neurotoxicity Syndrome (ICANS)

ICANS is a known and serious complication in patients who have received CAR-T therapy [9]. This has previously been referred to as CRES (CAR‑T‑cell‑related encephalopathy syndrome). ICANS is defined as: “a disorder characterized by a pathologic process involving the central nervous system following any immune therapy that results in the activation or engagement of endogenous or infused T cells and/or other immune effector cells. Symptoms or signs can be progressive and may include aphasia, altered level of consciousness, and impairment of cognitive skills, motor weakness, seizures, and cerebral oedema” [9].

Early symptoms may include:

- Headache
- Confusion
- Impaired handwriting
- Tremors
- Aphasia
- Encephalopathy

Advanced symptoms may include:

- Somnolence
- Seizures
- Cerebral edema
- Coma

The reported incidence is highly variable. The onset of neurotoxicity can coincide with CRS or be sequential to it, but can also occur as late as the third or fourth week following CAR-T cell infusion, after resolution of CRS. The management of ICANS involves the use of tocilizumab, steroids, anti-epileptics for seizures, urgent referral to and reviews by a neurologist, and possible early transfer to ICU for monitoring. Consideration should also be given to the use of prophylaxis with levetiracetam.

A 10-point toxicity score called ICE (Table 2) has been proposed and is based on the mini-mental state examination [9]. This is used in conjunction with assessment of consciousness level, intracranial pressure, evidence of seizures and assessment of motor function to give an overall grade. Frequent monitoring at least 8–12 hourly using the ICE screening tool can detect early signs of ICANS (Table 2). Changes in handwriting can be an early sign of ICANS, and a handwriting test is a key element of the ICE tool. The monitoring of handwriting is an important nursing role; any changes, however subtle, should be escalated to the medical team without delay.

Grading of ICANS is not adequately captured using the Common Terminology Criteria for Adverse Events [10] or other toxicity scoring models. A grading tool was devised by as part of the ASTCT guidelines and is reproduced in Table 3 [9]. In this system, the final ICANS grade is determined by the most severe event among the different domains. Treatment is according to the grade of ICANS and presence or absence of specific complications e.g. raised intracranial pressure or seizures, and is outlined in Tables 4, 5 and 6.

Assessment of ICANS in children can be a little more challenging, as the ICE tool may not be appropriate for younger children. The Cornell Assessment of Pediatric Delirium tool [11] is recommended for the assessment of ICANS in children under the age of 12 and is reproduced in Table 7. This tool may also be used in children over the age of 12 with developmental delays, and is validated to be used up to the age of 21. The score produced by this tool then forms part of the ASTCT ICANS consensus grading for children, which incorporates the other domains to indicate an overall grade (see Table 8).

## Hemophagocytic Lymphohistiocytosis (HLH)/Macrophage Activation Syndrome (MAS)

HLH/MAS, a rare complication of CRS, is difficult to diagnose [8]. It is estimated to occur in around 1% of patients who receive CAR-T cells [12]. It is a group of immune responses driven by hyper activation of macrophages and lymphocytes, lymphohistiocytic tissue infiltration and pro inflammatory cytokine production, resulting in multi-organ dysfunction [13]. Many of the symptoms of HLH/MAS overlap with CRS, such as high fevers and elevated markers (such as ferritin, cytokines and CRP); it is usually a manifestation of severe CRS [7], but can be difficult to distinguish from primary HLH or other conditions that mimic sepsis. For patients treated with CAR-T, the only reliable indicators for HLH/MAS are hemophagocytosis, hypofibrinogenemia and, probably, hypertriglyceridemia, as many of the other diagnostic features are observed during CAR-T mediated CRS. Treatment is with tocilizumab and/or high-dose steroids, but some patients may require more active immune suppression, such as etoposide or ciclosporin [12].

**Fig. 1:**
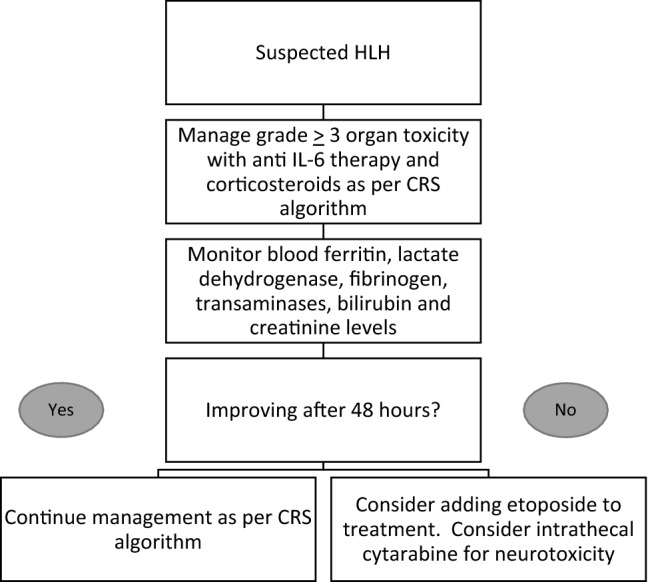
Recommendations for the management of CAR-T cell related Hemophagocytic Lymphohistiocytosis (HLH) /Macrophage Activation Syndrome (MAS) [12]

There are diagnostic criteria for pediatric HLH [13] and those for CAR-T related HLH/MAS have been suggested [12], (Table 9 and Fig. 1). The key nursing roles in the management of HLH/MAS are monitoring the patient for abnormalities in vital signs and communicating changes effectively to the medical team, to enable prompt decision making. There will likely be very close monitoring of the patient and potentially liaising with more than one medical team involved in the management, especially if the patient is admitted to the intensive care unit – which is likely in the event of HLH/MAS.

## Post Discharge Monitoring Following CAR-T

1. It is recommended that patients and care givers receive verbal and written information/education on:

a. The symptoms of CRS and serious neurologic adverse reactions

b. The need to report the symptoms to their treating team immediately

c. The need to remain in close proximity of the location where the CAR-T cells were received for at least 4 weeks following infusion.

2. Patients are advised not to drive for 8 weeks post infusion. This is due to the risk of delayed neurological toxicity.

## Long Term Follow Up

In addition to the risk of relapse from their primary hematological malignancy, CAR-T therapy recipients are at risk of developing new complications beyond the immediate weeks following cell infusion, such as hematological disorders, neurologic, autoimmune manifestations or second malignancies.

Hypogammaglobulinemia and B-cell aplasia are known effects of treatment with CAR-T cell as the CAR-T cells target an antigen on the surface of B-cells. The targeted cancer cells are killed, as are some or all of the healthy B-cells, which is known as an “on-target-off-tumor” effect. These patients are at sustained increased risk of infection due to B cell aplasia, and can sometimes require treatment with intravenous immunoglobulins as prophylaxis in cases of severe and/or prolonged infections [15].

The establishment of guidance for follow up plays a key role in the safe use of CAR-T, contributing to the cellular therapy community’s body of knowledge on the effects of this treatment which, in turn, informs our patient care. Regular assessment of quality of life using a validated multi-domain tool will generate further knowledge of the impact of illness and treatment on patients who have received this therapy. The results should be taken into consideration and used to inform care planning or modifying the care of the individual patient. Follow up should also be seen as an opportunity to impact on overall well-being, to reinforce appropriate health promotion messages, and to provide education and information to improve self-management.

## Scope of Follow-Up

In the setting of CAR-T therapy, the guidelines refer to long term follow-up (LTFU) monitoring, as that performed at the time points outlined in Table 10, and for the currently recommended duration of 15 years (commended by United States Food and Drug Administration and European Medicines Agency), considering that CAR-T cells qualify both as Gene Therapy Medicinal Products and Genetically Modified Organisms).

It is important to note that the recommendations in this document offer general guidance on LTFU following a CAR-T therapy, and observations should also consider product-specific characteristics, basic and translational knowledge generated in the clinical setting, and product-specific preclinical data.

Outcomes should be reported through EBMT specific forms for patients who have already been registered at infusion or, preferably, when apheresis is planned (this being important to evaluate the proportion of patients who cannot receive the drug product due to various circumstances arising during the manufacturing process).https://www.ebmt.org/sites/default/files/2019-01/25%20Cellular%20Therapy%20MED-A.pdf

Data should be submitted at the following 4 time points:

1. First report—Day 0: Use Day 0 Registration for Cell Therapy.

2. Second report—Day 100: Use Day 100 form for Cell Therapy.

3. Third report—6 Month Follow Up: Use the Follow Up form for Cell Therapy.

4. Fourth report—Annual Follow Up: Use the same Follow Up as in third report.

Centers should also comply with national regulations which, in some countries, have established national and/or disease-focused registries, and with product-specific requirements that appear in Post Authorization Safety Surveys (PASS) carried out by Marketing Authorization Holders (MAH), independently or collaboratively with continental registries, such as EBMT.

## Assessments and Evaluations

There are a range of assessments and evaluations to consider in follow-up, some of which are disease and/or product specific. Table 10 offers an illustration of the extent of possible tests to consider. This is not an exhaustive list and other investigations may be indicated in individual patients. However, it is important for the nurse to be aware of the range and extent of tests that the patient may require, in order to offer support with appointments, co-ordination of investigations and communication of results where appropriate, as well as escalation of patient reported concerns to the appropriate senior, when necessary.

There is no clear guidance in the literature for second malignancy screening, but this is an area of long-term concern which we are advised to monitor following treatment with a novel cellular therapy product. In the absence of guidance, the healthcare team should ensure that patients are receiving age-appropriate screening as per the general population for breast, cervical, prostate and bowel cancers, supported by education and information on regular breast and testicular self-assessment, skin awareness and sun protection, as well as health promotion to moderate risk factors such as obesity, smoking and alcohol intake.

## Conclusion

CAR-T therapy is a promising new treatment modality, but with significant toxicities. These guidelines serve as a framework for management, and are based on the current available evidence and experience. Nurses play a pivotal role at each key stage of the pathway from preparation, collection; infusion, post-infusion care and long term follow up.

## Figures and Tables

**Table 10:**
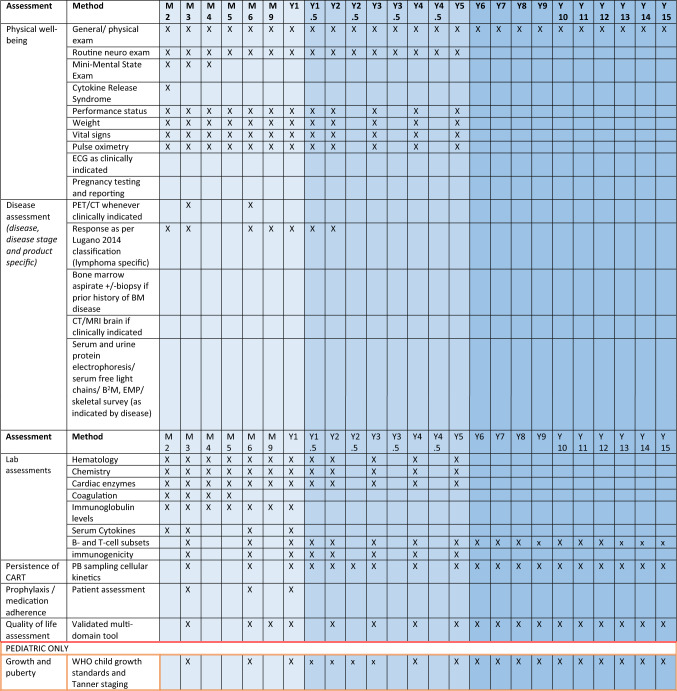
Range of possible assessments and evaluations (M2 to Y15)

## Data Availability

Not applicable.

## Appendix

See Tables 1, 2, 3, 4, 5, 6, 7, 8, 9, 10 and Fig. 1.

**Table 1:** American Society of Transplant and Cellular Therapy (ASTCT) consensus grading of Cytokine Release Syndrome [9]:

CRS Parameter*	Grade 1	Grade 2	Grade 3	Grade 4
Fever^#†^	Temperature ≥ 38 °C	Temperature ≥ 38 °C	Temperature ≥ 38 °C	Temperature ≥ 38 °C
	With either:			
Hypotension^#^	None	Not requiring vasopressors	Requiring one vasopressor with or without vasopressin	Requiring multiple vasopressors (excluding vasopressin)
	And/ or^‡^			
Hypoxia^#^	None	Requiring low-flow nasal cannula^	Requiring high-flow nasal cannula^, facemask, non-rebreather mask, or Venturi mask	Requiring positive pressure (eg: CPAP, BiPAP, intubation and mechanical ventilation)

#Not attributable to any other cause

†In patients who have CRS then receive tocilizumab or steroids, fever is no longer required to grade subsequent CRS severity

‡CRS grade is determined by the more severe event

^Low-flow nasal cannula is < 6 L/min and high-flow nasal cannula is > 6 L/min

*Organ toxicities associated with CRS may be graded according to Common Terminology Criteria for Adverse Events (CTCAE) v5.0 but they do not influence CRS grading

**Table 2:** ICE Tool [9]

Immune-Effector Cell-Associated Encephalopathy (ICE) Tool
Orientation: Orientation as to year, month, city, hospital: 4 points
Naming: Name 3 objects (e.g., Point to clock, pen, button): 3 points
Following commands: (e.g., ‘Show me 2 fingers’ or ‘Close your eyes and stick out your tongue’): 1 point
Writing: Ability to write a standard sentence (e.g., Our national bird is the bald eagle): 1 point
Attention: Count backwards from 100 by ten: 1 point

**Table 3:** Grading of neurotoxicity as per ASTCT consensus guidelines [9]

Neurotoxicity Domain	Grade 1	Grade 2	Grade 3	Grade 4
ICE Score	7–9	3–6	0–2	0 (Patient is unarousable and unable to perform ICE)
Depressed level of consciousness	Awakens spontaneously	Awakens to voice	Awakens only to tactile stimulus	Patient is unarousable or requires vigorous or repetitive tactile stimuli to arouse. Stupor or coma
Seizure	N/A	N/A	Any clinical seizure focal or generalised that resolves rapidly; or Non-convulsive seizures on EEG that resolve with intervention	Life-threatening prolonged seizure (> 5 min); or Repetitive clinical or electrical seizures without return to baseline in between
Motor findings	N/A	N/A	N/A	Deep focal motor weakness such as hemiparesis or paraparesis
Raised intracranial pressure / Cerebral oedema	N/A	N/A	Focal/local oedema on neuroimaging	Diffuse cerebral oedema on neuroimaging; Decerebrate or decorticate posturing; or Cranial nerve VI palsy; or Papilleodema; or Cushing's triad

**Table 4:** Treatment of Immune Effector Cell Associated Neurotoxicity Syndrome (ICANS) [12]

Grade	Treatment
1	Vigilant supportive care; aspiration precautions; intravenous (IV) hydration. Withhold oral intake of food, medicines, and fluids, and assess swallowing. Convert all oral medications and/or nutrition to IV if swallowing is impaired. Avoid medications that cause central nervous system depression. Assess for alternative causes of neurological dysfunction (e.g. metabolic, infection). Fundoscopic exam to assess for papilloedema Low doses of lorazepam (0.25–0.5 mg IV every 8 h) or haloperidol (0.5 mg IV every 6 h) can be used, with careful monitoring, for agitated patients. For patients with seizures or seizure like activity, start anticonvulsivants (first line levetiracetam 500 mg iv then 1000 mg 12 hourly). Patients may require alternative/additional anti-convulsivants if ineffective. For treatment of uncontrolled seizures see below For patients with uncontrolled seizures or no improvement in 24–48 h arrange for neurology consultation, EEG, MRI of the brain with and without contrast; diagnostic lumbar puncture with measurement of opening pressure; MRI spine if the patient has focal peripheral neurological deficits; CT scan of the brain can be performed if MRI of the brain is not feasible Consider dexamethasone^1^ 10 mg three times per day po/iv if early onset (within 3 days of CAR-T treatment) For anti-IL-6 therapy with tocilizumab 8 mg/kg* IV, per protocol if ICANS is associated with concurrent CRS
2	Supportive care and neurological work-up as described for grade 1 ICANS Tocilizumab 8 mg/kg IV if associated with concurrent CRS Dexamethasone^1^ 10 mg IV every 8–12 h (more frequent if onset less than 3 days following CAR-T therapy) Neurology consultation, EEG, MRI of brain with and without contrast; diagnostic lumbar puncture with measurement of opening pressure; MRI spine if the patient has focal peripheral neurological deficits; CT scan of brain can be performed if MRI of brain is not feasible Consider transferring patient to intensive-care unit if ICANS associated with grade ≥ 2 CRS
3	Supportive care and neurological work-up as indicated for grade 1 ICANS Transfer to intensive care unit is recommended Anti-IL-6 therapy if associated with concurrent CRS, as described for grade 2 ICANS and if not administered previously Dexamethasone^1^ 20 mg iv every 8 h corticosteroids, until improvement to grade 1 ICANS and then taper Stage 1 or 2 papilloedema with CSF opening pressure < 20 mmHg should be treated as below Neurology consult and consider repeat neuroimaging (CT or MRI), EEG and if patient has persistent grade ≥ 3 ICANS
4	Supportive care and neurological work-up as outlined for grade 1 ICANS Intensive care unit monitoring mandatory; consider mechanical ventilation for airway protection Anti-IL-6 therapy as described for grade 3 ICANS Dexamethasone^1^ 20 mg IV four times per day. Consider methylprednisolone 2 mg/kg/day in divided doses (can increase to 500-1000 mg/day) if no response Neurology consult and consider repeat neuroimaging (CT or MRI), EEG and if patient has persistent grade ≥ 3 ICANS For convulsive status epilepticus, treat as below Stage ≥ 3 papilloedema, with a CSF opening pressure ≥ 20 mmHg or cerebral oedema requires specific management and neurological ITU transfer (see below)

^1^Where dexamethasone is started it should be continued for a minimum of 48 h (5–7 days for grade 3–4 disease) with taper or until symptoms resolve. Higher and more frequent dosing are recommended for early onset toxicity, refractory seizures and reduced conscious level

**Table 5:** Management of status epilepticus [12]

Grade	Treatment
Non convulsive	Assess airway, breathing, and circulation; check blood glucose Lorazepam 0.5 mg intravenously (IV), with additional 0.5 mg IV every 5 min, as needed, up to a total of 2 mg to control electrographical seizures Levetiracetam 500 mg IV bolus, as well as maintenance doses If seizures persist, transfer to intensive-care unit add additional anti-epileptic drugs – to discuss with neurology/intensive care team Maintenance doses after resolution of non-convulsive status epilepticus are as follows: lorazepam 0.5 mg IV every 8 h for three doses; levetiracetam 1,000 mg IV every 12 h
Convulsive	Assess airway, breathing, and circulation; check blood glucose Transfer to intensive care unit Lorazepam 2 mg IV, with additional 2 mg IV to a total of 4 mg to control seizures Levetiracetam 500 mg IV bolus, as well as maintenance doses If seizures persist add additional anti-epileptic drugs – to discuss with neurology/intensive care team Maintenance doses after resolution of convulsive status epilepticus are: lorazepam 0.5 mg IV every 8 h for three doses; levetiracetam 1,000 mg IV every 12 h; phenobarbital 1–3 mg/kg IV every 12 h Continuous EEG monitoring should be performed, if seizures are refractory to treatment
Patients with uncontrolled seizures should be discussed with neurology and may require transfer to neurology ITU	

**Table 6:** Management of raised intracranial pressure [12]

Grade	Treatment
Early^1^	Consider acetazolamide 1,000 mg intravenously (IV), followed by 250–1,000 mg IV every 12 h (adjust dose based on renal function and acid–base balance)
Advanced^2^	Use high-dose corticosteroids with methylprednisolone IV 1 g/day, as recommended for grade 4 ICANS Elevate head end of the patient’s bed to an angle of 30 degrees Consider hyperventilation to achieve target partial pressure of arterial carbon dioxide (PaCO_2_) of 28–30 mmHg Hyperosmolar therapy with either mannitol or hypertonic saline Consider CSF drainage Intracranial pressure monitoring Consider neurosurgery options

^1^Stage 1–2 papilloedema or CSF pressure < 20 cm CSF

^2^Stage 4–5 papilloedema or radiological evidence of cerebral oedema or CSF pressure ≥ 20 cm CSF. Patients with significant raised intracranial pressure require transfer to neurology intensive care for monitoring and treatment

**Table 7:** Encephalopathy Assessment for Children Age < 12 Years Using the Cornell Assessment of Pediatric Delirium [11]

Answer the following based on interactions with the child over the course of the shift:					
	Never, 4	Rarely, 3	Sometimes, 2	Often, 1	Always, 0
1. Does the child make eye contact with the caregiver?					
2. Are the child's actions purposeful?					
3. Is the child aware of his/her surroundings?					
4. Does the child communicate needs and wants?					
	Never, 0	Rarely, 1	Sometimes, 2	Often, 3	Always, 4
5. Is the child restless?					
6. Is the child inconsolable?					
7. Is the child underactive; very little movement while awake?					
8. Does it take the child a long time to respond to interactions?					

For patients age 1–2 years, the following serve as guidelines to the corresponding questions:

1. Holds gaze, prefers primary parent, looks at speaker

2. Reaches and manipulates objects, tries to change position, if mobile may try to get up

3. Prefers primary parent, upset when separated from preferred caregivers. Comforted by familiar objects (i.e., blanket or stuffed animal)

4. Uses single words or signs

5. No sustained calm state

6. Not soothed by usual comforting actions, e.g., singing, holding, talking, and reading

7. Little if any play, efforts to sit up, pull up, and if mobile crawl or walk around

8. Not following simple directions. If verbal, not engaging in simple dialog with words or jargon

**Table 8:** ASTCT ICANS Consensus Grading for Children [9]

Neurotoxicity Domain	Grade 1	Grade 2	Grade 3	Grade 4
ICE score for children age ≥ 12 years*	7–9	3–6	0–2	0 (patient is unarousable and unable to perform ICE)
CAPD score for children age < 12 years	1–8	1–8	_9	Unable to perform CAPD
Depressed level of consciousness†	Awakens spontaneously	Awakens to voice	Awakens only to tactile stimulus	Unarousable or requires vigorous or repetitive tactile stimuli to arouse; stupor or coma
Seizure (any age)	N/A	N/A	Any clinical seizure focal or generalized that resolves rapidly or nonconvulsive seizures on EEG that resolve with intervention	Life-threatening prolonged seizure (> 5 min); or repetitive clinical or electrical seizures without return to baseline in between
Motor weakness (any age) ¥	N/A	N/A	N/A	Deep focal motor weakness, such as hemiparesis or paraparesis
Elevated ICP/ cerebral edema (any age)	N/A	N/A	Focal/local edema on Neuroimaging §	Decerebrate or decorticate posturing, cranial nerve VI palsy, papilledema, Cushing's triad, or signs of diffuse cerebral edema on neuroimaging

ICANS grade is determined by the most severe event (ICE or CAPD score, level of consciousness, seizure, motor findings, raised ICP/cerebral edema) not attributable to any other cause. Baseline CAPD score should be considered before attributing to ICANS

* A patient with an ICE score of 0 may be classified as grade 3 ICANS if awake with global aphasia, but a patient with an ICE score of 0 may be classified as grade 4 ICANS if unarousable

† Depressed level of consciousness should be attributable to no other cause (eg, no sedating medication)

¥ Tremors and myoclonus associated with immune effector cell therapies may be graded according to CTCAE v5.0, but they do not influence ICANS grading

§ Intracranial hemorrhage with or without associated edema is not considered a neurotoxicity feature and is excluded from ICANS grading. It may be graded according to CTCAE v5.0

**Table 9:** Diagnostic criteria for CAR-T cell related Hemophagocytic Lymphohistiocytosis (HLH) /Macrophage Activation Syndrome (MAS) [12]

Diagnostic criteria for CAR-T cell related HLH/MAS:
A patient might have HLH/MAS if he/she has a peak serum ferritin level of > 10,000 ng/ml during the cytokine release syndrome phase of CAR-T cell therapy (typically the first 5 days after cell infusion) and subsequently develops any two of the following:
Grade ≥ 3 increase in serum bilirubin, aspartate aminotransferase or alanine aminotransferase levels*
Grade ≥ 3 oliguria or increase in serum creatinine levels *
Presence of hemophagocytosis in bone marrow or organs based on histopathological assessment of cell morphology and/or CD68 immunohistochemistry

* Grading as per Common Terminology Criteria for Adverse Events, version 4.03
